# Implant-Based Breast Reconstruction after Mastectomy, from the Subpectoral to the Prepectoral Approach: An Evidence-Based Change of Mind?

**DOI:** 10.3390/jcm11113079

**Published:** 2022-05-30

**Authors:** Andrea Weinzierl, Daniel Schmauss, Davide Brucato, Yves Harder

**Affiliations:** 1 Institute for Clinical & Experimental Surgery, Saarland University, 66421 Homburg, Germany; andrea.weinzierl@uks.eu; 2 Department of Plastic Surgery and Hand Surgery, University Hospital Zurich, 8091 Zurich, Switzerland; 3 Department of Plastic, Reconstructive and Aesthetic Surgery, Ospedale Regionale di Lugano, Ente Ospedaliero Cantonale (EOC), 6900 Lugano, Switzerland; schmauss.daniel@gmail.com (D.S.); davide.brucato@eoc.ch (D.B.); 4 Faculty of Biomedical Sciences, Università della Svizzera Italiana, 6900 Lugano, Switzerland

**Keywords:** prepectoral breast reconstruction, synthetic mesh, biologic mesh, implant-based breast reconstruction, mastectomy, hybrid breast reconstruction

## Abstract

Over the last years, prepectoral implant-based breast reconstruction has undergone a renaissance due to several technical advancements regarding mastectomy techniques and surgical approaches for the placement and soft tissue coverage of silicone implants. Initially abandoned due to the high incidence of complications, such as capsular contraction, implant extrusion, and poor aesthetic outcome, the effective prevention of these types of complications led to the prepectoral technique coming back in style for the ease of implant placement and the conservation of the pectoralis muscle function. Additional advantages such as a decrease of postoperative pain, animation deformity, and operative time contribute to the steady gain in popularity. This review aims to summarize the factors influencing the trend towards prepectoral implant-based breast reconstruction and to discuss the challenges and prospects related to this operative approach.

## 1. Introduction

Attempts to surgically remove breast cancer lesions date back thousands of years. Even the oldest known surgical document, the Edwin Smith Egyptian papyrus, contains reasonings on how to treat breast tumors [1]. Even though early attempts were mostly contained to rapid excisions and/or cauterization due to the lack of anesthesia, usually brutal experimental procedures continued to be performed despite the excessive disfigurement, morbidity, and mortality [2]. In 1804, Japanese surgeon Seishu Hanaoka used a self-concocted anesthetic mixture to perform what some believe to be the world’s first procedure under general anesthesia, a mastectomy [3]. Only 90 years later, Halsted published his landmark paper “*The results of operations for the cure of cancer of the breast performed at the Johns Hopkins Hospital from June 1889, to January 1894*” [4]. In what is today known as Halsted’s radical mastectomy, all suspected tissues, including the pectoralis major muscle, were resected *en bloc* to prevent the spread of the breast cancer and to cure the disease. Later in his career, Halsted recommended an even more extensive dissection, including, for instance, the supraclavicular lymph nodes [5]. His procedure continued to be the standard of care up until the 1940s, when the additional use of radiation therapy became more widespread. Techniques such as the modified radical mastectomy by Madden [6], which spared the pectoralis muscle, or the simple mastectomy showed similar oncological outcomes to Halsted’s mastectomy when combined with radiation therapy [2]. Advances in cancer biology, a better understanding of the pathophysiology, and consistent screening methods resulted in the development of the concept of breast conservation surgery, as well as substantially changed approaches to mastectomy. Freeman described the subcutaneous or skin preserving mastectomy for benign lesions of the breast as early as 1962 [7], though the technique was not used for the treatment of breast cancer until decades later [8]. In addition, nipple sparing mastectomy techniques were popularized over time, as they allowed for an excellent aesthetic outcome close to the original aspect of the breast [9].

**Figure 1:**
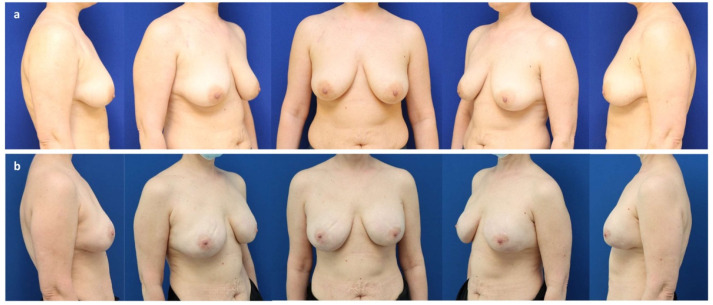
(**a**) A 41-year-old patient with invasive-ductal carcinoma (pT2 pN1a (3/3) M0) of the left breast and a BRCA-1 mutation after neo-adjuvant chemotherapy with mild volume asymmetry and breast ptosis grade II (Regnault classification). (**b**) Six years after the bilateral nipple sparing mastectomy and immediate subpectoral implant-based breast reconstruction (Motiva Ergonomix^®^ ERSD 475 cc, Establishment Labs Motiva, Alajuela, Costa Rica) and ADM (Strattice™ tissue matrix, LifeCell Corporation; Branchburg, NJ, USA) through a periareolar access with lateral extension to correct the ptosis and adjuvant radiotherapy of the left breast. Note the rippling of the right breast (upper inner quadrant), skin retraction, and capsular contraction grade II (Baker classification) of the left breast.

**Figure 2:**
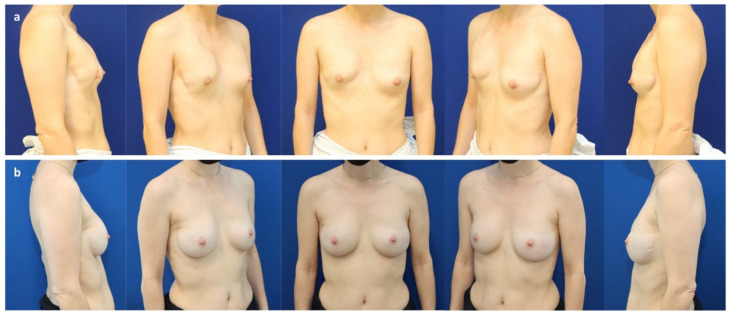
(**a**) A 45-year-old patient after a bilateral nipple-sparing mastectomy through a lateral access for invasive-ductal carcinoma (pT2 pN0 M0) of the right breast and a prophylactic mastectomy of the left breast after immediate subpectoral implant-based reconstruction elsewhere. Note the capsular contraction grade IV (Baker classification) and implant displacement on the right. (**b**) Six years after bilateral partial capsulectomy, reconstruction of the subpectoral implant pocket using a resorbable synthetic mesh (Vicryl^®^, Ethicon, Cincinnati, OH, USA), and implant exchange (Motiva Ergonomix^®^ ERSD 300 cc).

**Figure 3:**
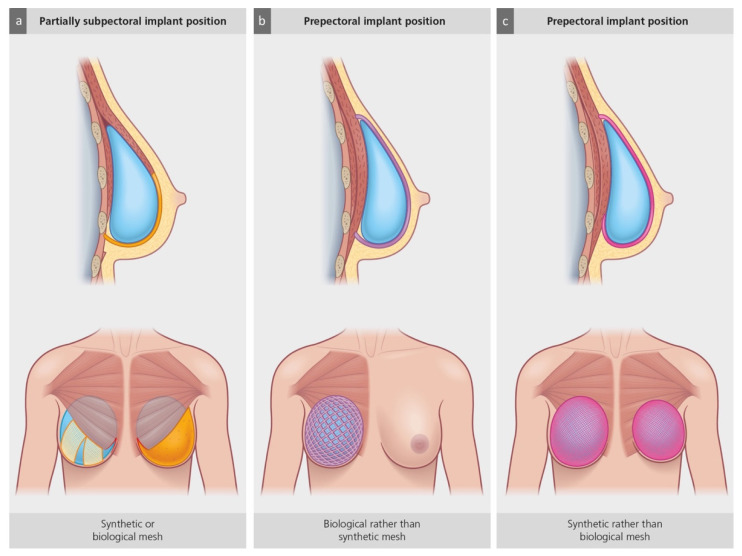
Schematic drawing of three different clinical situations during implant-based breast reconstruction (BR). (**a**) Subpectoral placement of an expander or implant requires the partial detachment of the greater pectoral muscle with partial medial disinsertion. Absence of fixation using synthetic mesh strips (right hemithorax) or biologic meshes (acellular dermal matrix; ADM; left hemithorax) will result in cranial muscle retraction. (**b**) Prepectoral expander or implant placement in patients with thin mastectomy flaps may benefit from thickened adipocutaneous implant coverage, due to ADM or synthetic meshes with tissue-integrative potential. (**c**) Prepectoral implant placement in patients with a mismatch between the large footprint of the breast after a mastectomy (e.g., breast hypertrophy) or a wide implant pocket in revisional breast surgery (e.g., down-sizing of breast implant volume) and implant size. When using a smaller implant, these patients may benefit from implant positioning and fixation using synthetic, pocket-shaped meshes rather than ADMs.

Parallel to this development, reconstructive procedures continued to evolve, including the search for suitable implants. Starting with the transplantation of a lipoma to fill the defect left by a partial mastectomy [10], ivory, glass, rubber, cartilage, wool, polyethylene chips, and even sponges have been used as breast implants [11]. In the 1960s, Cronin and Gerow reported the use of a silicone gel breast implant for breast augmentation, and this was soon after followed by the initial use of silicone implants for breast reconstruction [12,13]. The initial pre-pectoral placement was soon abandoned, due to massive rates of capsular contraction, implant extrusion, infection, and poor aesthetic outcome (Figure 1) [14]. The subsequent shift to the subpectoral plane offered an increased coverage of the implant and the effective prevention of some of these complications [14]. Newer operative techniques led to initially pleasing results [15]. However, subpectoral implant placement in turn is frequently associated with chronic muscle related pain, muscle spasms or contractions, animation deformity of the reconstructed breast, reduced physical mobility of the upper extremity, and eventually a reduction in physical strength of the patient (Figure 2) [16,17]. Therefore, prepectoral implant-based breast reconstruction (IBBR) has undergone a renaissance, utilizing various technical advancements to control and prevent the initially encountered challenges of the technique (Supplementary Video S1). Among these advancements are the continued optimization of mastectomy techniques, advances in radiation therapy, the use of alloplastic adjuncts and autologous fat grafts, as well as new implant designs (Figure 3).

With the present narrative review, we want to give an overview of the factors that have contributed to the current trend towards prepectoral breast reconstruction and discuss the related chances and challenges, as well as illustrate them with appropriate clinical cases.

## 2. Current Mastectomy Techniques and Their Implications for Implant-Based Breast Reconstruction

The continuously growing share of women desiring breast reconstruction after mastectomy has markedly contributed to the rise in IBBR, as autologous procedures, including microvascular flaps, are generally less available [18]. The trend towards bilateral procedures has also added to this effect [19]. Such bilateral procedures are commonly performed as either a contralateral prophylactic mastectomy in patients with unilateral cancer, or as a bilateral prophylactic mastectomy in women with an increased breast cancer risk. While a clear risk reduction for the development of breast cancer could be demonstrated in high-risk populations, such as carriers of genetic mutations [20,21], an increasing number of patients that do not carry gene mutations still decide to undergo a contralateral prophylactic mastectomy anyways [22], without clear evidence for a substantial survival benefit [23,24,25,26,27]. In these cases, however, the use of prepectoral IBBR seems particularly attractive, as the minimally invasive muscle sparing approach appeals to the generally young and both functionally, as well as aesthetically demanding patient cohort [28,29]. Moreover, bilateral implant placement improves the perception of breast symmetry [27], and unlike autologous flap-based reconstruction, it does not result in a collateral damage due to tissue harvesting.

Besides a clear shift in the quantity, it is above all the technique and quality of performed mastectomies that has changed over time. Surgical mastectomy has gradually undergone a paradigm shift from the “maximum tolerable” to the “minimum effective” treatment [30]. Conservative surgical approaches, such as skin-sparing mastectomies (SSM) and nipple-sparing mastectomies (NSM), have become the standard of care, whenever the resection of the entire mammary gland is oncologically warranted or desired by the patient, both in the curative and prophylactic setting. By preserving as much as possible of the soft tissue envelope of the breast, these techniques attribute a much greater value to a women’s body image and well-being after treatment and ultimately represent a new attitude towards breast cancer therapy. While SSM has been universally recognized to be an oncologically safe and effective treatment option [31], NSM was initially scrutinized due to the small amount of residual ductal tissue behind the nipple–areola complex (NAC), considered to be at risk of an occult NAC involvement and subsequent local recurrence [32,33]. However, recent studies report few or no nipple recurrences and overall survival rates of up to 98–99%, thus supporting the safety of this procedure [34]. Nonetheless, histopathological analyses of retroareolar biopsies have become part of the standard procedure [35]. Even though patients experience a significant loss of sensation [36], the preservation of the breast envelope is associated with an improved postoperative body image and sexual function and has ultimately led to the clinical implementation of SSM and NSM techniques whenever possible [37,38]. Moreover, future technical advancements will continue to change the standards of care in breast cancer surgery. Robotic NSM, for instance, has emerged as a novel approach that promises to push the efforts to conserve the breast envelope even further [39,40,41]. Even though its safety and feasibility are still being evaluated, the initial meta-analysis did not find significant differences in complication rates when comparing robotic NSM to traditional NSM [42].

If conservative mastectomy is indicated, several patient-related factors, such as body-mass-index, breast volume and grade of ptosis, active smoking habits and co-morbidities such as diabetes, as well as disease-related factors, including the need for adjuvant therapy, influence the surgical course of action and the possibility of prepectoral IBBR. Patients with hypertrophic and ptotic breasts remain a challenging subgroup for prepectoral IBBR, as they have a higher risk of developing complications such as NAC-necrosis or delayed wound healing [43,44]. Skin reducing mastectomy (SRM) has emerged as a way to utilize the skin excess for additional soft tissue coverage [43,45]. Skin reduction is achieved by using common mastopexy incision patterns, with the Wise pattern being used most frequently. This results in a de-epithelialized inferior adipocutaneous flap that is used for additional soft tissue coverage of the inferior implant pole [46]. In selected cases, these flaps can also be used to stabilize the implant in its subcutaneous pocket. In addition, new incision patterns continue to be described with the intention to perfect this technique [47,48]. Another interesting approach is the technique of “preshaping” [49,50,51]. For this approach, mastopexy or reduction mastoplasty is performed as a first step, followed by risk-reducing NSM and prepectoral IBBR a few months later.

Once the mammary gland excision has been performed, it is part of the competence of the breast surgeon to adequately evaluate the intraoperative situation regarding thickness, dimensions, and perfusion of the mastectomy flap, as adequate soft tissue coverage is essential for successful prepectoral IBBR. Should the intraoperative perfusion of the mastectomy flap remain dubious, conversion to a subpectoral approach, at least temporarily, may decrease the likelihood of mastectomy skin flap necrosis and reconstructive failure [52]. If prepectoral placement of a tissue expander or implant was carried out, the used surgical approach should also influence subsequent treatment decisions. For instance, if minor skin flap necrosis occurs, aggressive management with early debridement may be necessary to avoid exposure of the device or any implanted materials, as the pectoralis muscle cannot function as a barrier between the mastectomy flap and the surgical pocket [52].

## 3. The Use of Biologic and Synthetic Meshes in Prepectoral IBBR

**Figure 4:**
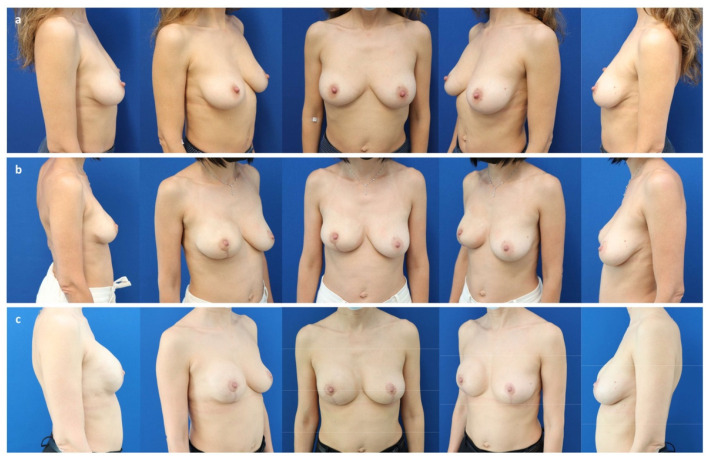
(**a**) A 43-year-old patient with multifocal invasive-ductal carcinoma (pT1c pN1mi M0) of the right breast. (**b**) Six months after a nipple sparing mastectomy through a lateral hemi-areolar access with vertical extension and prepectoral expander placement (Motiva Flora^®^ XMF-58 440 cc) and ADM (SurgiMend^®^ PRS meshed, Integra LifeSciences, Princeton NJ, USA) use. (**c**) One year after expander exchange for a definitive implant (Motiva Ergonomix^®^ ERSD 300 cc) and autologous fat grafting to the mastectomy flap (140 cc) and mastopexy of the left breast (“auto-augmentation”).

Another important factor that has influenced the trend towards prepectoral breast reconstruction is the increasing use of biologic meshes (acellular dermal matrices = ADMs) and synthetic meshes. Initially popularized for direct-to-implant (DTI) reconstructions [53], they have become increasingly popular in both single- and two-stage IBBR. Over time, different types of alloplastic adjuncts have become available, including ADMs derived from human, bovine, and porcine dermis, as well as absorbable, partially absorbable, and non-absorbable synthetic meshes [54]. Even though they bear a certain endogenous potential for complications, such as infection, seroma formation, and red breast syndrome [55,56], ADMs and meshes became an important player in the prevention of implant-associated complications in prepectoral IBBR [57]. Their use ensures an improved implant placement and soft tissue coverage by wrapping them around the entire implant or using them to cover its front side (Figure 4). The implant can thus be fixed to the pectoralis major muscle or its fascia without dissecting the muscle, reducing postoperative pain and facilitating a fast recovery [58]. The use of ADMs and meshes also decreases the pressure on the caudal mastectomy flap, preventing ischemic wound complications during the acute postoperative phase or a “bottoming out” of the implant over time. This improved fixation is particularly beneficial when un-textured implants are used, as they do not adhere to the surrounding soft tissue over time and therefore progressively expand the skin. Furthermore, the use of alloplastic adjuncts can compensate for an unfavorable mismatch between the size of the tissue pocket and the implant’s width after mastectomy or in revisional surgery after IBBR. In this case, the internal fixation of the implant further helps avoid complications, such as the lateral displacement of the implant in the supine position.

Furthermore, the use of ADMs and meshes makes IBBR in irradiated patients a feasible option [59]. Despite the added costs and potentially higher complication rates, surgeons advocate the use of ADMs in irradiated or otherwise complex patients, who might particularly benefit from their use [60,61]. Several studies have demonstrated a significantly lower rate of capsular contraction due to reduced chronic inflammation and subsequent tissue elastosis in this therapeutic setting [60,62,63,64,65,66].

Alloplastic adjuncts should, however, not be used without careful consideration. Instead, the surgeon should implant as little foreign material as possible without compromising the desired stabilization and coverage of the implant. In this context, a retrospective analysis showed that thicker ADMs are associated with an increased incidence of seroma, infection, or skin necrosis [67]. This is probably due to a slower neovascularization and thus, a later integration of the ADM into the surrounding tissues of the mastectomy flap [68]. It does, therefore, remain dubitable whether the prepectoral position should still be used in borderline cases such as particularly slim patients or under very thin mastectomy flaps, creating an unfavorable ratio between patient tissue and matrix to be integrated with a disproportionately high risk of complications.

## 4. Autologous Fat Grafting and Hybrid Prepectoral Reconstruction

**Figure 5:**
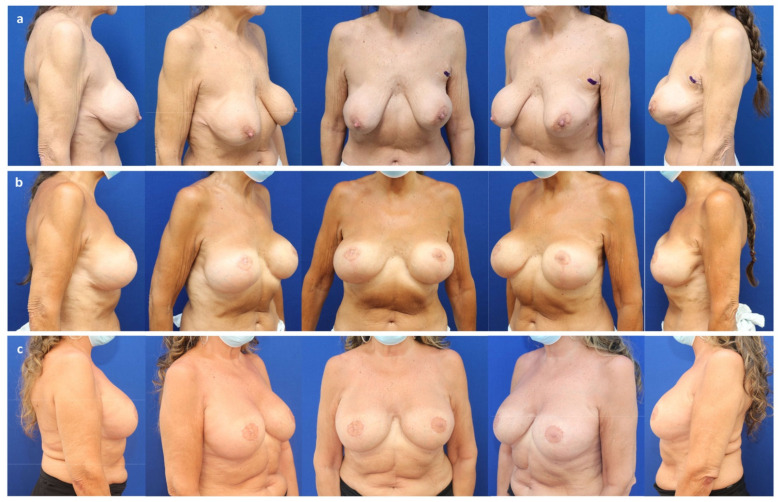
(**a**) A 62-year-old patient with persistent in situ ductal carcinoma (pTis pN0 M0) of the left breast after tumorectomy, as well as bilateral ptosis grade II (Regnault classification) and capsular contracture grade IV (Baker) 30 years after prepectoral augmentation mastoplasty. (**b**) Six months after a bilateral skin reducing mastectomy, free nipple graft, and prepectoral expander placement (Mentor CPX4 550 cc, Mentor Worldwide LLC, Irvine, CA, USA). (**c**) Two years after a bilateral expander exchange for a definitive implant (Motiva Ergonomix^®^ ERSD 575 cc), synthetic pocket-like mesh (TiLOOP^®^ Bra Pocket, pfm medical AG, Cologne, Germany), and autologous fat grafting to the mastectomy flaps (210 cc/breast).

An indirect contribution of ADM-use to the trend towards prepectoral IBBR is the fact that biological meshes do not only increase the thickness of the thin mastectomy flaps, but they can also serve as a well vascularized recipient tissue for subsequent autologous fat grafting (AFG) after their successful integration (Figure 5). The oncological safety of AFG after breast cancer has been demonstrated in several clinical trials, including long term follow up studies [69,70]. AFG thickens the soft tissue coverage of the inserted implant and, thus, improves the aesthetic outcome of IBBR by reducing complications such as implant rippling or contour irregularities. In fact, several studies have shown that this procedure significantly improves patient satisfaction after breast cancer surgery and IBBR [71,72,73].

**Figure 6:**
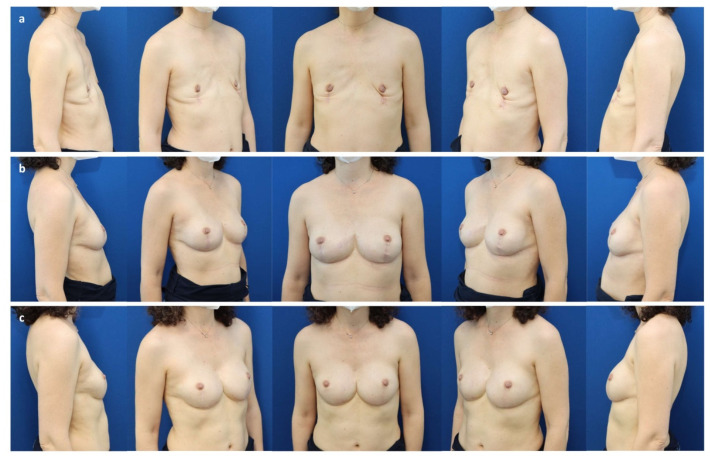
(**a**) A 44-year-old patient with mild structural deformity of the anterior chest wall and state after bilateral implant-removal for infection following a nipple-sparing mastectomy and immediate subpectoral implant-based reconstruction elsewhere for multifocal invasive-ductal carcinoma (pT2 pN 1a (3/3) M0) associated with an extended in situ component of the right breast and BRCA-2 mutation. (**b**) Three months after completed expansion (Motiva Flora^®^ XMF-58 440 cc) and before the first session of autologous fat grafting. (**c**) One year after the expander-to-implant based prepectoral breast reconstruction and two sessions of autologous fat grafting (70–90 cc per breast: reversed expansion, “hybrid breast reconstruction”; Motiva Ergonomix^®^ ERSM 275 cc).

Due to the beneficial effects of AFG, some surgeons have begun to postulate hybrid or composite concepts that combine prepectoral DTI reconstruction with AFG during the same operative procedure [74]. A more gradual surgical approach is the so called “reverse expansion”, where the placed tissue expander is drained in a stepwise fashion once the desired skin expansion is reached, and the removed volume is replaced with several AFG sessions (Figure 6) [75]. By markedly augmenting the subcutaneous fat compartment, a more desirable ratio between smaller implant volume and thicker soft-tissue coverage can be achieved. In addition to the increased quantity of tissue, AFGs have also been shown to improve its quality [76]. This aspect of AFG is particularly beneficial for patients that have previously been irradiated [73]. For these patients, the risk of early complications, reconstruction failure, and poor aesthetic results can reach up to 50% [77]. Carrying out AFG, either before the tissue expander is substituted with the definitive implant or even before the prophylactic mastectomy is performed, significantly improved surgical outcomes [78,79,80].

Ultimately, no amount of soft tissue coverage can completely eradicate complications associated with the use of silicone implants, but breast surgeons may be able to increase and prolong the durability of prepectoral IBBR, especially in irradiated patients prone to implant failure, and therefore reduce the need for implant-related revisional surgery. However, general implant-related complications such as breast asymmetry remain a concern regarding the long-term outcome of prepectoral IBBR [81]. While it is an appropriate surgical approach for patients that lack sufficient tissue or refuse flap-based breast reconstruction [82], it may be prudent to consider that the cost of repeated AFG sessions, implant placement, and substitutions rivals the cost of flap-based autologous breast reconstruction without the benefit of being a lifetime solution.

## 5. Radiation Therapy and Prepectoral IBBR

**Figure 7:**
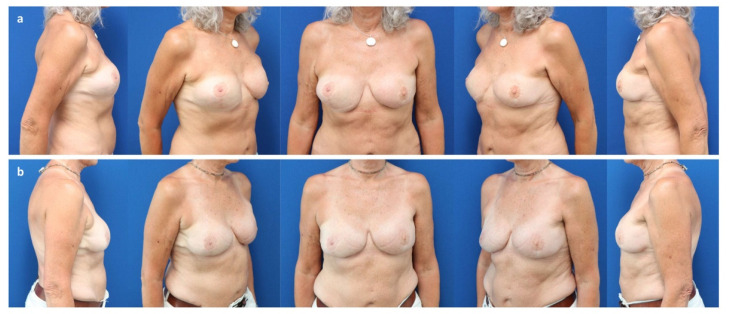
(**a**) A 59-year-old patient after a bilateral skin reducing mastectomy for invasive lobular carcinoma (pT3 pN2 (10/10) M0) of the left breast and immediate subpectoral implant-based reconstruction elsewhere, followed by adjuvant radiotherapy. Exchange of implants with microvascular flaps from the abdomen (DIEP). Salvage of the breast pocket with an implant on the right for flap failure. Note the caudal implant displacement and asymmetry of breast shape. (**b**) Three years after a pocket change from subpectoral to prepectoral, exchange of the implant (Motiva Ergonomix^®^ ERSM 400 cc), reconstruction of the inframammary fold, and autologous fat grafting to the mastectomy flap (220 cc).

Even though radiation therapy is an integral part of breast cancer treatment, the use of postmastectomy radiotherapy considerably complicates breast reconstruction, in particular when implants are used (Figure 7) [83]. In addition, a considerable part of patients undergoing a mastectomy has previously undergone breast conserving treatment consisting of tumorectomy and adjuvant radiotherapy. However, IBBR in irradiated patients has become more feasible with the introduction of ADMs and the use of AFG to counteract radiation damage and complications such as capsular contracture or implant extrusion [59,79,80,84]. Interestingly, prepectoral implant placement has been shown to be beneficial in this context. Subpectorally placed implants exhibited a significantly higher rate of capsular contractures when compared to prepectoral implants in irradiated patients [85]. In addition, the observed cases of capsular contractures were markedly more pronounced in subpectorally placed implants when graded according to the Baker classification [85].

Another development that may further ease the combination of radiation therapy and IBBR is the emerging concept of preoperative “neo-adjuvant” radiation protocols [86,87,88,89]. Combining preoperative radiation therapy with immediate reconstruction could bear several advantages, as it avoids the direct irradiation of the implant and prevents the need for secondary reconstruction after tissue expander placement. Ideally, the initial consolidation and wound healing can thus take place before any chronic radiation damage, such as fibrosis or microangiopathy, reaches its full extend [90]. Initial studies do not show relevant differences in overall survival when comparing preoperative and postoperative radiation [91]. Even though such protocols rely on a close interdisciplinary collaboration and on adhering to a strict perioperative treatment sequence, this optimization may further decrease the incidence of surgical complications in breast reconstruction.

## 6. Further Technical Developments

Other technical advances have contributed to the trend towards prepectoral IBBR in smaller, yet interesting ways. Several techniques have been described to increase the survival rate and tissue quality of mastectomy flaps; for instance, there is the use of local heat preconditioning [92,93] or hyperbaric oxygen therapy [94]. Furthermore, the development and clinical implication of intraoperative indocyanine green angiography (ICG) for the real time visualization of tissue perfusion has been another crucial step toward perfecting prepectoral IBBR [95]. The immediate intraoperative assessment of the perfusion of the mastectomy skin flap to guide excision of inadequately perfused areas has since translated to improved clinical outcomes. By preventing necrosis or wound dehiscence, ICG has proven to be a helpful tool in reconstructive breast surgery, though large randomized clinical trials are still warranted to confirm its effectiveness [96]. Other issues that need to be addressed in the future are the currently missing standardization and the possible overprediction of necrosis [97].

**Figure 8:**
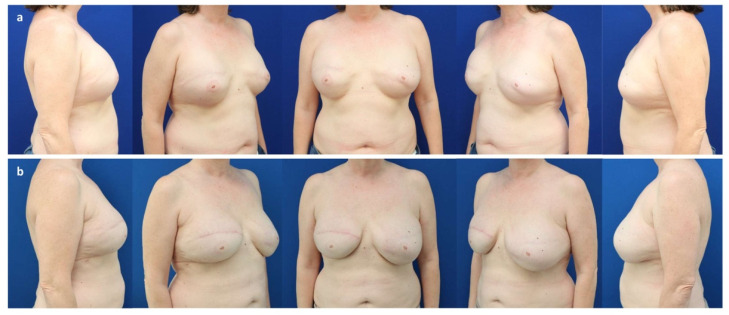
(**a**) A 54-year-old patient with state after a bilateral mastectomy for multifocal invasive ductal carcinoma (pT3 pN2 (10/10) M0) of the right breast and prophylactic mastectomy of the left breast, as well as immediate subpectoral expander-to-implant-based reconstruction elsewhere, followed by adjuvant immuno-chemotherapy. Note the bilateral capsular contracture (Baker classification grade IV). (**b**) Two years after a pocket change from subpectoral to prepectoral, exchange of the implant (Motiva Ergonomix^®^ ERSD 575 cc), and autologous fat grafting to the mastectomy flaps (220 cc/side) without mesh-support. Note bottoming out of the left.

Nowadays, surgeons can also choose from a much larger range of implants to best suit the patient’s needs. Textured implants, which fuse better with the surrounding tissue, prevent a “bottoming out” of the implant. Although the risk is very low, the risk of implant-associated anaplastic large cell lymphoma (BIA-ALCL) must be considered and discussed with the patient, but they may still be indicated in specific situations [98]. Besides textured implants, newer lightweight implants are an additional option to reduce pressure on the caudal mastectomy flap [99]. Especially when large implants are necessary, as often is the case in reconstructive breast surgery, this may be beneficial for the initial wound healing process after surgery and long-term prevention of bottoming out (Figure 8).

## 7. Conclusions

Taken together, prepectoral implant placement after mastectomy has become a valid surgical alternative for breast reconstruction. By maintaining the breast envelope and increasing soft tissue coverage, complications such as implant extrusion and capsular contracture can effectively be decreased, even in irradiated patients. Short-term benefits such as a rapid recovery and maintained pectoralis muscle function contribute to the patient’s psychosocial well-being and high satisfaction with the reconstructive result. Though many long-term complications related to subpectoral implant placement, including breast animation and impaired muscle function, are effectively avoided, general implant-related complications, such as breast asymmetry, remain a concern for prepectoral IBBR. It does, however, provide good to excellent aesthetic and functional results for patients that cannot or do not wish to undergo autologous breast reconstruction, for instance because they lack sufficient tissue.

In conclusion, the collective learning curve of prepectoral IBBR has not reached a plateau yet, though massive strides have been made in the last years. Besides the shared experiences of breast surgeons worldwide, large scale clinical trials will continue to provide crucial information and opportunities to further improve the technique. Surgeons should therefore strive to incorporate new developments into their clinical routines as they emerge to provide patients with the best possible care.

## Data Availability

Not applicable.

## Supplementary Materials

The following supporting information can be downloaded at: https://www.mdpi.com/article/10.3390/jcm11113079/s1, Supplementary Video S1: Prepectoral breast reconstruction.
